# Giant urethral caruncle resembling urethral prolapse causing outflow obstruction

**DOI:** 10.1016/j.eucr.2021.101783

**Published:** 2021-07-15

**Authors:** Alfa Putri Meutia, Kevin Yonathan, Fina Widia

**Affiliations:** a Division of Urogynecology and Reconstruction Department of Obstetrics and Gynecology, Faculty of Medicine, Universitas Indonesia, Dr. Cipto Mangunkusumo National General Hospital, Jakarta, Indonesia; b Urogynecology Clinic, Bunda General Hospital, Jakarta; c Faculty of Medicine, Universitas Indonesia, Dr. Cipto Mangunkusumo National General Hospital, Jakarta, Indonesia; d Department of Urology, Faculty of Medicine, Universitas Indonesia, Dr. Cipto Mangunkusumo National General Hospital, Jakarta, Indonesia

**Keywords:** Urethral caruncle, Pelvic organ prolapse, Urethral disease

## Abstract

Urethral caruncle is a rare condition primarily affecting elderly. Sometimes, it resembles urethral prolapse or malignancies. It can even cause outflow obstruction or urinary retention. A case of 83-years-old woman with urinary retention since a week prior was presented. Physical examination revealed a bulging mass originating from posterior lip of external urethral meatus. Due to the size, it caused outflow obstruction. The whole mass was excised. Histology examination reported the mass as urethral caruncle. Urethral caruncle is a common problem in elderly which rarely cause outflow obstruction. A thorough examination is required to distinguish it from malignancies and other disorders.

## Introduction

Urethral caruncle is a benign lesion, usually originating from the posterior part of urethra. It is one of the most common benign tumor of female urethra, especially in elderly. Due to its low incidence and benign feature, there is no wide scale research regarding its etiology. However, it is thought to be related to low estrogen level in postmenopausal women.^1^ Usually, it is asymptomatic and found incidentally. However, there are cases of urethral caruncle causing symptoms such as dysuria or even urinary retention due to its enormous size.^2^

This study aims to present a case of giant urethral caruncle resembling urethral prolapse, which caused outflow obstruction.

## Case

An 83-year-old P6A0 postmenopausal woman came to our urogynecology center with chief complaint of urinary retention which exacerbated since a week ago. She said that her urine was only dripping even after straining herself. This symptom had occurred since a year ago. However, she thought that it was one of the symptoms of aging, thus she never went for medical examination. She also complained of a bulging sensation felt at her vagina.

There was no complaint of defecating problem nor abnormal vaginal discharge prior to the visit. Her last menstruation was more than 30 years ago. However, she had seen some blood stains on her underwear over the past week. She was a widow and her last sexual activity was more than 20 years ago.

She had 6 child, all of which was born vaginally. She had never known the birth weight of her babies. Previously, she had never used any kind of contraceptive. There are no complaints of fatigue nor rapid loss of weight. Her prior medical condition was also unremarkable.

**Fig. 1:**
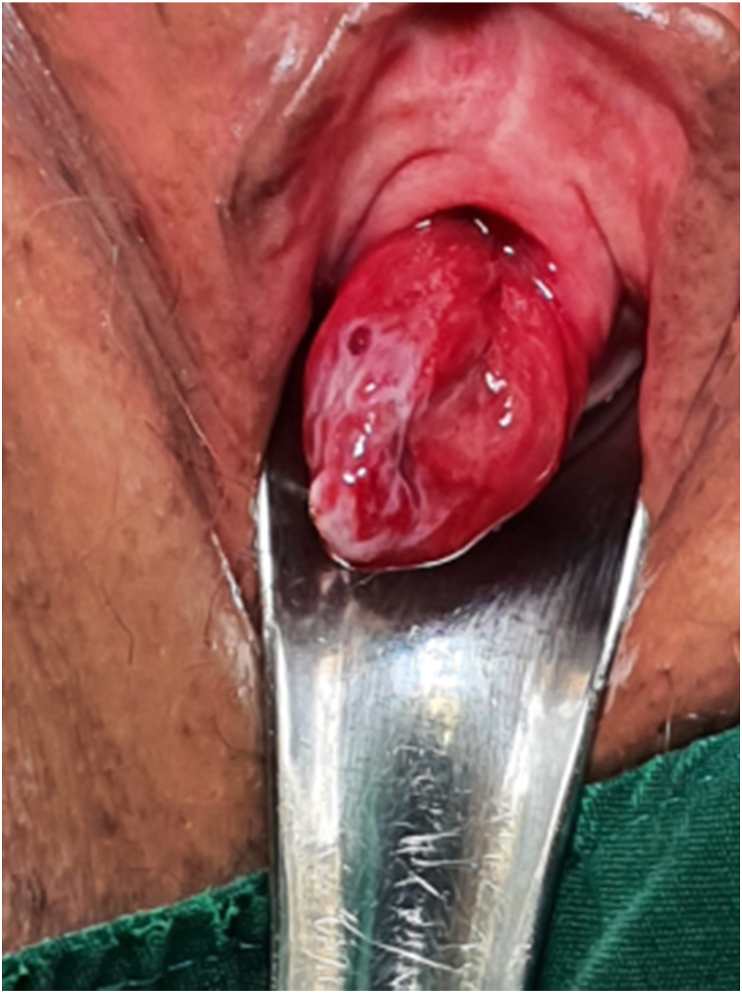
Urethral caruncle during pelvic examination.

Patients vital sign was stable at the time of arrival. Physical examination revealed bulging pedunculated red mass originating from posterior lip of the external urethral meatus. Its dimension was 25 mm × 25 mm, was soft and fixated on palpation. At a glance, it was thought to be urethral prolapse. However, additional examination showed that the prolapse only occurred on the posterior lip of the urethra. It was then thought to be a urethral caruncle. (Fig. 1).

Physical examination revealed no other pathology on bladder and kidney. Complete blood count, urine examination, and uroflowmetry were done following the meeting. Her blood and urine result was within normal limit while her uroflowmetry result revealed intermittent flow due to abdominal straining with reduced average flow rate without any residual urine.

Due to the disturbing symptoms, the patient was scheduled to have total surgical excision of the urethral caruncle on the same day. Following preparation and acquisition of informed consent, total excision under general anesthesia was performed. Following the urethral dilatation, urethrocystoscopy was also performed with normal result.

The whole mass was totally resected, while the urethral meatus was then sutured with 3–0 absorbable sutures and 18 French (F) Foley silicon urinary catheter was placed in order to prevent stenosis and help urine drainage. The patient was then discharged while the mass was sent for histology examination.

On the seventh day following the surgery, there were no complications found and the catheter was removed accordingly. The histopathologic result reported the mass as hyperplastic urothelium overlying inflamed stroma. The result was consistent with a urethral caruncle. Follow up uroflowmetry on the 14th day showed normal result. There was no sign of residual symptoms or recurrence after 6 months of follow up.

## Discussion

Elderly women are prone to having gynecological problem due to various anatomical and hormonal changes, such as sexual dysfunction, pelvic floor disorders, and malignancies.^3^ One of the overlooked problems arising from hormone deficiency in women is urethral caruncle. Urethral caruncle is a common disorder primarily presents in postmenopausal women, although sometimes it can be found on prepubertal women.^1^ It usually presents as benign, pedunculated, and highly vascular mass at the urethral meatus.^1^ Generally, urethral caruncle is small in size and asymptomatic at diagnosis. However, some patients will complain of dysuria or infrequent bleeding.^4^

There are some confusions regarding the difference between urethral caruncle and urethral prolapse. Urethral caruncle is a benign mass of the urethral meatus, arising from epithelial hyperplasia, subepithelial inflammation, and fibrosis, sometimes mimicking a neoplasm.^4^ It usually originates from the posterior lip of the urethra. Urethral prolapse, on the other hand, is induced by excessive pressure, resulting of circumferential eversion of the mucosa.^5^ Hence, the extrusion in urethral prolapse will be circumferential. Additionally, there is no inflammation occurring in urethral prolapse. In our case, the extrusion was only occurring at the posterior lip of the urethra. However, it is big enough to cause urethral outflow obstruction.

Although it is commonly asymptomatic, urethral caruncle can present as a gigantic mass, causing either outflow obstruction or even urinary retention.^2^ This phenomenon needs more prompt treatment. If the caruncle has been treated, the symptoms will subsequently subsided.

Initial treatment for urethral caruncle consists of topical estrogen and oral anti-inflammatory treatment.^1^ However, we chose to perform total excision due to the severity of symptoms at the time of diagnosis and the abnormal size of the mass. This approach was also chosen in similar cases.^2^^,^^4^

Although previous study has stated that urethral caruncle does not have any association with increased risk of malignancy, thorough examination is required in order to distinguish urethral caruncle from other disorders.^4^ There were some cases of suspected urethral caruncle that would eventually represent malignancy, such as urothelial carcinoma.^4^ Thus, histopathological examination following excision is required, especially on elderly patients with large mass such as ours.

## Conclusion

Urethral caruncle is a common gynecological problem especially in elderly. Giant urethral caruncle can cause urinary outflow obstruction and even urinary retention. Thorough examination is required to identify and treat it accordingly.

## Approval of the research protocol

The protocol for this research project has been approved by a suitably constituted Ethics Committee of the institution and it conforms to the provisions of the Declaration of Helsinki. Committee of Research Ethics Committee of Faculty of Medicine, University of Indonesia, approval No. KET-285/UN2.F1/ETIK/PPM.00.02/2019. All informed consent was obtained from the subject(s) and/or guardian(s).

## Informed consent

Written informed consent was obtained from the patient for anonymized patient information to be published in this article.

## Previous presentation

Authors declare that the study has never been presented on scientific presentation before.

## Funding source

This research did not receive any specific grant from funding agencies in the public, commercial, or not-for-profit sectors.

## Registry and registration number of the study

Not applicable.

## CRediT authorship contribution statement

**Alfa Putri Meutia:** Conceptualization, Formal analysis, Investigation, Resources, Writing – original draft, Supervision, Funding acquisition. **Kevin Yonathan:** Methodology, Software, Writing – original draft, Validation, Project administration. **Fina Widia:** Validation, Investigation, Supervision.

## Declaration of competing interest

Authors declare that there is no conflict of interest in this study.
